# Application of toxicology in silico methods for prediction of acute toxicity (LD_50_) for Novichoks

**DOI:** 10.1007/s00204-023-03507-2

**Published:** 2023-05-05

**Authors:** Maciej Noga, Agata Michalska, Kamil Jurowski

**Affiliations:** 1 Department of Regulatory and Forensic Toxicology, Institute of Medical Expertises in Łódź, Ul. Aleksandrowska 67/93, 91-205 Łódź, Poland; 2 Institute of Medical Expertises in Łódź, Ul. Aleksandrowska 67/93, 91-205 Łódź, Poland; 3 Laboratory of Innovative Toxicological Research and Analyzes, Institute of Medical Studies, Medical College, Rzeszów University, Al. Mjr. W. Kopisto 2a, 35-959 Rzeszów, Poland

**Keywords:** Novichoks, Organophosphate, Nerve agents, Acute toxicity, Toxicology in silico

## Abstract

Novichoks represent the fourth generation of chemical warfare agents with paralytic and convulsive effects, produced clandestinely during the Cold War by the Soviet Union. This novel class of organophosphate compounds is characterised by severe toxicity, which, for example, we have already experienced three times (Salisbury, Amesbury, and Navalny's case) as a society. Then the public debate about the true nature of Novichoks began, realising the importance of examining the properties, especially the toxicological aspects of these compounds. The updated Chemical Warfare Agents list registers over 10,000 compounds as candidate structures for Novichoks. Consequently, conducting experimental research for each of them would be a huge challenge. Additionally, due to the enormous risk of contact with hazardous Novichoks, in silico assessments were applied to estimate their toxicity safely. In silico toxicology provides a means of identifying hazards of compounds before synthesis, helping to fill gaps and guide risk minimisation strategies. A new approach to toxicology testing first considers the prediction of toxicological parameters, eliminating unnecessary animal studies. This new generation risk assessment (NGRA) can meet the modern requirements of toxicological research. The present study explains, using QSAR models, the acute toxicity of the Novichoks studied (*n* = 17). The results indicate that the toxicity of Novichoks varies. The deadliest turned out to be A-232, followed by A-230 and A-234. On the other hand, the "Iranian" Novichok and C01-A038 compounds turned out to be the least toxic. Developing reliable in silico methods to predict various parameters is essential to prepare for the upcoming use of Novichoks.

## Introduction

**Fig. 1:**
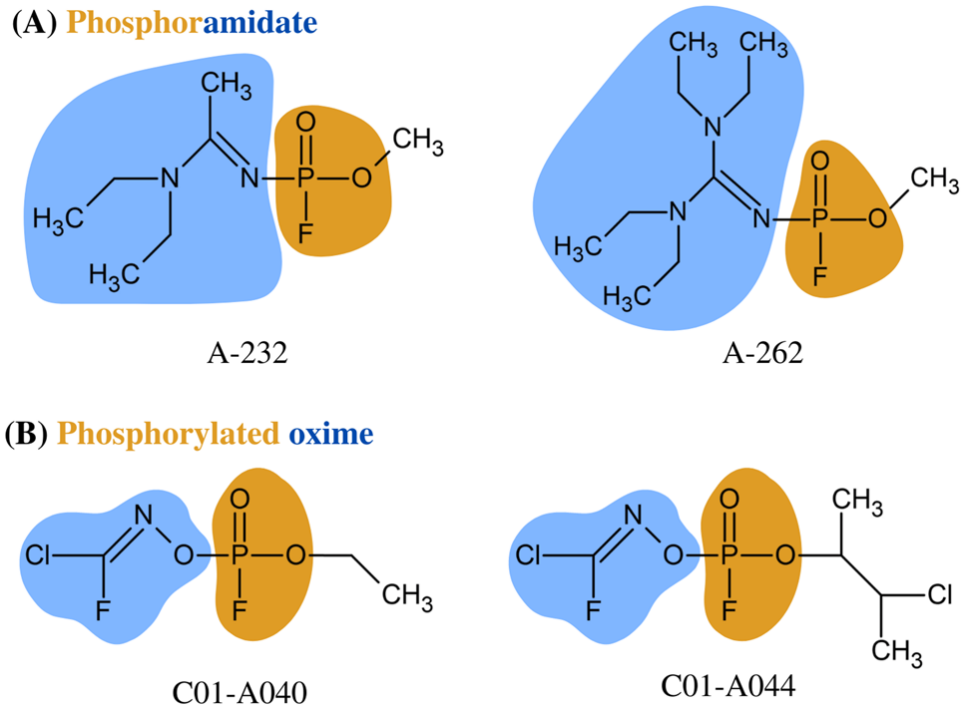
Postulated chemical structures of Novichoks: **A** Mirzayanov’s A-232 and A-262 as phosphoramidate, **B** Ellison’s C01-A040 and C01-A045 as phosphorylated oxime

Novichoks (Russian: oвиoк, ‘newcomer’), referred to as nerve agents of series A (NA), pose a group of chemical warfare agents with a paralysing and convulsive effect (Noga and Jurowski 2023). Assume that Novichok compounds are unique organophosphates (OPs) containing a dihaloformaldoxime moiety (Watson et al. 2015). It is assumed that Vil S. Mirzayanov revealed the first information about A-series compounds in his book "State Secrets: An Inside Chronicle of Russia's Chemical Weapons Programme" (Mirzayanov 2008). Two possible Novichok structures have been postulated, although it is unclear which one is more reliable. Mirzayanov published the first as phosphoramides (Fig. 1A). On the contrary, the second structure was proposed by Hoenig (2007) and Ellison (2007) as phosphorylated oximes (Fig. 1B). Furthermore, a group of Iranian scientists synthesised an additional Novichok structure in the laboratory (Hosseini et al. 2016). The probable chemical structures of the Novichoks and SMILES notation are presented in Table 1.

**Table 1:**
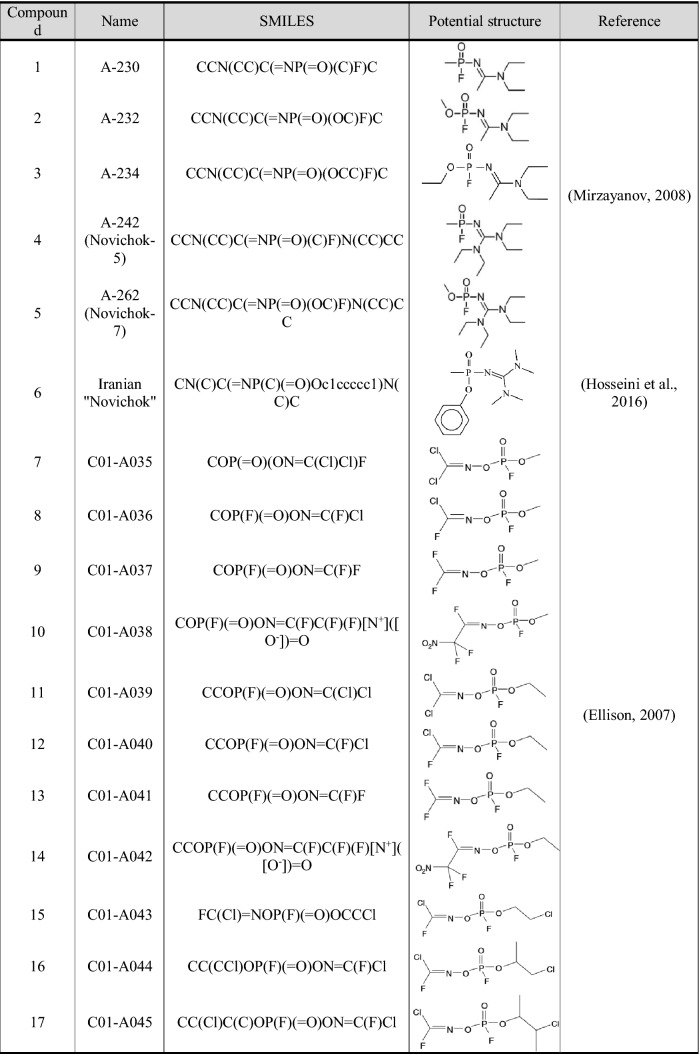
Possible chemical structures of studied Novichok nerve agents

The original design intent of the Novichoks was to circumvent the Chemical Weapons Convention (CWC) list and be undetected using standard North Atlantic Treaty Organisation (NATO) chemical detection equipment (Nepovimova and Kuca 2020). One of the probable mechanisms of the toxic effect of Novichok compounds is irreversible binding to acetylcholinesterase (AChE) and inhibition of hydrolysis of the neurotransmitter acetylcholine (ACh) to acetate and choline (Chai et al. 2018). Overstimulation of cholinergic receptors caused by the accumulation of ACh in the synaptic cleft as a result of inhibition of AChE leads, depending on the route, dose and time of exposure, to the manifestation of several toxic symptoms through three types of reactions: muscarinic, nicotinic and central nervous system (CNS) (Korabecny et al. 2014; Kloske and Witkiewicz 2019; Kloske 2020).

So far, little is still known about Novichoks, and more data must be completed. One is knowledge about the threat level of these compounds, that is, their toxicity. We have already witnessed the "show" of the enormous toxic potential of Novichoks three times. The first two cases of use of these nerve agents took place in 2020 in Salisbury and Amesbury (UK) and started a public debate that made everyone aware of the dangerous nature of these compounds (Bhakhoa et al. 2019; Haslam et al. 2022). The third example of using A series nerve agents was the case of Navalny's acute poisoning in 2020 during a domestic flight in Russia. Following the results of clinical and laboratory studies, the use of a cholinesterase inhibitor was identified. This incident is critical because it is the only published clinical study on Novichok poisoning treatment, which proved the ineffectiveness of obidoxime reactivation and the effectiveness of butyrylcholinesterase therapy (Steindl et al. 2021).

The above examples indicate the presence of Novichoks in public spaces and confirm the enormous threat and severe poisoning effects of these compounds in series A. Therefore, from the point of view of social security, it is crucial to study their properties, especially their toxicological aspects. The toxicity of Novichoks as a hypothetical group of nerve agents should be a critical national issue. There are many problematic issues, and the fundamental questions from a toxicological point of view are; What threat do these substances pose when in contact with humans? Exposure to what dose of these hazardous compounds is lethal? Do the A-series compounds exceed the toxicity of previous generations of NAs (-V and -G)? To answer these questions, it is essential to determine the toxicity of Novichoks. Therefore, estimating the acute toxicity of these chemicals (LD_50_) in humans will be necessary to solve these problems. It should be noted that LD_50_ is based on crude endpoints (harmful effects) that estimate the average response (for statistical reasons) of a single exposure, in contrast to the value of the absence of the effect of multiple doses; it only documents when a compound causes death in animals. Although it appears to be a primary toxicological parameter, it is no longer usually experimentally determined in many situations (toxicological risk assessment usually requires other parameters such as the level of non-observed adverse effects, NOAEL). Furthermore, there is no correlation between LD_50_ and other compounds (e.g., biological activity, developmental, and reproductive toxicity (DART)). It differs significantly from a 'true' starting point for obtaining health values. Furthermore, LD_50_ is not generated based on the principles of replacement, reduction, and refinement of animal use and welfare (3R), which are principles aimed at minimising the use of animals in toxicity tests when applicable (Faria et al. 2016). However, given the specificity of this topic and the severe gap in determining such a fundamental toxicological parameter for Novichok (just a few works related to this crucial issue (Bolt and Hengstler 2022), there is a great need for this type of research. Therefore, to fulfil the modern requirements for toxicological research of toxicology of the twenty-first century and to consider the next-generation risk assessment (NGRA) (Pallocca et al. 2022) with a new approach to toxicity testing (Leist et al. 2008) (i.e., taking into account the prediction of toxicological parameters first), it is necessary to first apply in silico toxicology methods to eliminate unnecessary animal studies. Researching this parameter is essential to determine the accurate level of risk that Novichoks may pose. However, surprisingly little attention has been paid to this fundamental issue (Hartung 2009; Krewski et al. 2020).

Studies on Novichoks are rare and have only recently begun to emerge (Imrit et al. 2020; Bolt and Hengstler 2022). Taking into account the three cases of chemical attacks involving novel nerve agents, it is no doubt that determining the toxicological aspects of these hazardous substances is essential, but also tricky because of their high reactivity and toxicity; as organophosphorus compounds they are treated differently in toxicology than other poisons, for example, in the Cramer classification (Kroes et al. 2004). Recognising the vulnerability to the threat of terrorist activity, the most desirable and justified approach in this situation seems to be the use of in silico toxicological tools. Furthermore, because such hazardous substances are not available, the only way to assess this possibility is to use in silico tools. Such methods are desirable and necessary to predict the acute toxicity (LD_50_) of Novichoks.

**Fig. 2:**
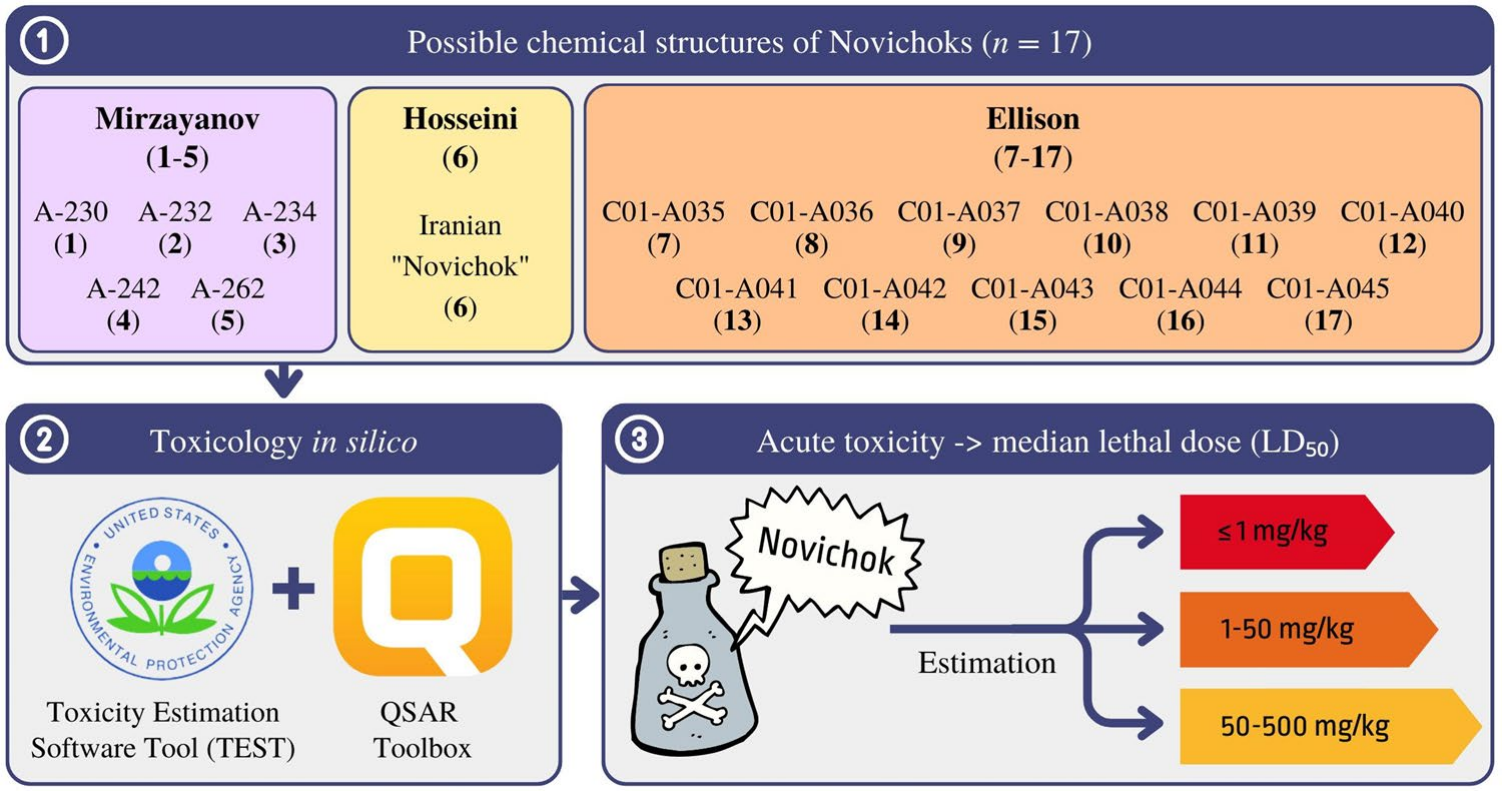
Flow chart that displays the general concept of median lethal dose estimation process applying in silico toxicology tools

Only a few studies on the application of computational/QSAR methods to study molecular aspects of Novichoks are available in the scientific literature (Nepovimova and Kuca 2018; Bhakhoa et al. 2019; Carlsen 2019; Franca et al. 2019; Jeong and Choi 2019; Harvey et al. 2020; Wang et al. 2021); however, they were never an exhaustive study of all known Novichoks. The rationale for conducting this study is the lack of primary data on Novichoks in the scientific literature (Bolt and Hengstler 2022). Reference is made to existing reports for only a few examples of these hazardous substances. To predict acute toxicity (LD_50_ for rats), we used models included in the software: QSAR Toolbox and Toxicity Estimation Software Tool (TEST) (Kleandrova et al. 2015). A general flow chart showing the acute toxicity parameter estimation process is presented in Fig. 2.

## Methods

### TEST

In silico studies were performed using the Toxicity Estimation Software Tool (TEST), an open-source application developed by the US EPA. Toxicity Estimation Software Tool (ver. 5.1.2 and ver. 4.2.1) comprises several models assessing acute toxicity thresholds by reading across structural analogues or multivariate regression. The models are built on hundreds of structural, constitutional, connectivity, shape, topological, molecular distance, fragments, and electrotopological property descriptors. The programme demands only SMILES (Simplified Molecular Line Input System) or CAS numbers, as inputs quickly evaluate chemical toxicity. The TEST software is trained on the endpoint from the EPA ECOTOX database (US EPA 2022). Every read-across or regression model has a specific applicability domain. The software offers an estimated LD_50_ threshold based on each model prediction and a Consensus average of the component models. TEST assesses acute toxicity (endpoint: oral rat LD_50_) using four QSAR methodologies (Martin et al. 2008):

- Hierarchical method: The toxicity for a particular query compound is estimated using the weighted average of the predictions from various models. The different models are achieved using Ward’s method to divide the training set into a series of similar structural clusters.
- FDA method (only ver. 4.2.1): The prediction for each test chemical is made using a new model that fits the chemicals most similar to the test compound. Each model is generated at runtime.
- Nearest-neighbour method: The predicted toxicity is estimated by averaging the three chemicals in training set with the closest similarity to the test compound.
- Consensus method: The predicted toxicity is estimated by taking an average of the predicted toxicities from each of the above QSAR methodologies (considering each method's applicability domain).

The Consensus result was reported as the most reliable estimate provided by the TEST software (Melnikov et al. 2016); therefore, we used it to estimate the acute toxicity of Novichok. In addition to the above method, the FDA method included in the TEST was also used to verify the reliability and compare the values with the results published by Carlsen (2019). Table 2 summarises the pros and cons of the available QSAR methodologies.

**Table 2:** Advantages and disadvantages of the QSAR methodology in TEST software (US EPA 2022)

Method	Advantages	Disadvantages
Hierarchical clustering	Produce more reliable predictions since predictions are made from multiple models	Cannot provide external estimates of toxicity for compounds in the training set
FDA	Generate a new model based on the closest analogues to test chemical Provides an external prediction of toxicity	Predictions sometimes take longer because it needs to generate a new model each time
Nearest neighbor	Provides a quick estimate of toxicity Allows to determine structural analogues for a given test compound Always provides an external prediction of toxicity	Does not use a QSAR model to correlate the differences between the test compound and the nearest neighbors Shown to achieve the worst prediction results during external validation
Consensus	Shown to achieve the best prediction results during external validation	Cannot provide external estimates of toxicity for compounds in the training set

### QSAR

We supported in silico analyses using the QSAR Toolbox ver. 4.5 standalone software application, which the OECD Organisation recommends for Economic Cooperation and Development. The QSAR Toolbox, developed by OASIS in collaboration with the OECD and the European Chemicals Agency (ECHA), is an application to evaluate the potential hazards of chemicals with in silico models to facilitate the practical application of (Q)SAR approaches in regulatory contexts by governments and industry, and to improve their regulatory acceptance (OECD and ECHA 2021). Data gaps are filled through the following flexible workflow in which compound categories are built, and incomplete data are estimated by read-across or applying local QSARs. In addition to read-across and trend analysis, the Toolbox includes numerous databases of experimental results. The calculated endpoint of this research was acute toxicity (LD_50_).

#### Estimation QSAR

Acute toxicity was estimated using QSAR Toolbox software by manual categorisation and data gap-filling method. Additionally, using the TEST software, this estimation was used to verify the accuracy of the previously calculated value. The target endpoint was defined in the following order: Human health hazards, acute toxicity, LD_50_ (endpoint), Oral (Route of administration) and rats (test organisms/species). The categorisation was defined as Repeated Dose (Hess); only in this grouping option is the Organophosphate category (organophosphorus compounds are treated differently in toxicology than other poisons, i.e. Cramer classification (Kroes et al. 2004)). The read data were selected only for the initially targeted endpoint. The read-through method for "qualitative" endpoints was used to fill data gaps. The scale/unit used to estimate acute toxicity (LD_50_) was chosen in (mg/kg). The rationale for selecting this scale/unit is that it offers the most considerable amount of chemicals and available converted data. Then, a subcategorisation was used to exclude structurally different prediction compounds from the investigated Novichoks. Individual subcategories were made for each chemical. The initial stage of subcategorisation for the targeted nerve agents had a particular common scheme. The option "Structure similarity" was used to remove dissimilar structures, and the option "US-EPA New Chemical Categories" and "Aquatic toxicity classification by ECOSAR" was used to remove selected analogues. The accepted predictions for Novichok compounds have been compiled in a table for the entire toxicity result section.

## Results

Acute toxicity, represented as the median lethal dose (LD_50_) of the Novichoks investigated (*n* = 17), was estimated using two software: QSAR Toolbox (ver. 4.5) and TEST. In the case of the latter tool, two versions have been used; the older one (ver. 4.2.1) contains the FDA model, which is missing in the newer one (ver. 5.1.2), where the Consensus model was used. The software calculates the LD_50_ values for oral administration to rats. The extrapolation from animal to human (rat-to-human) was based on toxicity values conversed following the guidelines for converting doses between animals and humans based on body surface area. Rat doses were converted to equivalent human doses by dividing the rat dose by 6.2 (Nair and Jacob 2016). The calculated median lethal dose values for the oral administration of Novichoks and human-converted LD_50_ values are provided in Table 3.

**Table 3:** Rat and human oral LD_50_ values calculated using the TEST and QSAR Toolbox software

Compound	Name	Rat oral LD50 (mg/kg bw)			Human oral LD50 (mg/kg bw)		
		TEST Consensus method	TEST FDA method	QSAR Toolbox	TEST Consensus method	TEST FDA method	QSAR Toolbox
1	A-230	2.14	9.59	3.09	0.35	1.55	0.50
2	A-232	1.31	3.52	2.53	0.21	0.57	0.41
3	A-234	3.57	4.43	3.91	0.58	0.71	0.63
4	A-242 (Novichok-5)	92.81	3.04	55.9	14.97	0.49	9.02
5	A-262 (Novichok-7)	276.26	45.56	141.7	44.56	7.35	22.85
6	Iranian "Novichok"	1109.56	596.17	810.3	178.96	96.16	130.69
7	C01-A035	206.51	1082.59	263.8	33.31	174.61	42.55
8	C01-A036	124.61	427.72	193.1	20.10	68.99	31.15
9	C01-A037	266.37	813.51	324	42.96	131.21	52.26
10	C01-A038	1922.26	2099.13	1030	310.04	338.57	166.13
11	C01-A039	35.39	34.44	48.6	5.71	5.55	7.84
12	C01-A040	26.05	4.19	15.4	4.20	0.68	2.48
13	C01-A041	49.11	95.16	58.5	7.92	15.35	9.44
14	C01-A042	206.76	27.17	129.9	33.35	4.38	20.95
15	C01-A043	16.47	6.21	12.5	2.66	1.00	2.02
16	C01-A044	39.72	31.23	45.7	6.41	5.04	7.37
17	C01-A045	243.78	96.01	191.8	39.32	15.49	30.94

In the case of estimated oral doses of LD_50_ for rats using the recommended Consensus method included in the TEST software and then converted doses for humans, the most perilous Novichok was A-232 (**2**), whose value was 0.21 mg/kg bw. Nerve agents indicated slightly higher median lethal doses: A-230 (**1**) 0.35 mg/kg bw and A-234 (**3**) 0.58 mg/kg bw. The remaining Novichok structures proposed by Mirzayanov (**4**–**5**) appeared to be weaker by almost two orders of magnitude than the compounds mentioned above (**1**–**3**). The ‘Iranian’ Novichok (**6**) and one of the structures proposed by Ellison C01-A038 (**10**) were the lowest potent of each compound studied, reaching the following values:

178.96 mg/kg bw and 310.04 mg/kg bw. The most lethal nerve agent among Ellison structures was C01-A043 (**15**), with a value of 2.66 mg/kg bw, although one order of magnitude weaker than the structure (**2**). The compounds (**11**–**13** and **16**) pose a slightly lower threat than the previously mentioned Novichok (**15**) and reach the following values: 5.71; 4.20; 7.92, and 6.41 mg/kg bw. Other Novichoks structures postulated by Ellison (**7**–**9**, **14** and **17**) showed values similar (from 20.10 to 42.96 mg/kg bw) to organophosphorus compounds (**4**–**5**).

The FDA model implemented in the TEST software calculated the LD_50_ values for each nerve agent studied. The results for only two compounds (**10–11**) were consistent compared to those estimated using the Consensus model. According to the FDA method, compound A-242 (**4**) had the lowest LD_50_ value, 0.49 mg/kg bw. Novichok A-232 (**1**) reached a slightly higher value of 0.57 mg/kg bw; with the Consensus method, it became the most hazardous compound. Interestingly, the next somewhat less toxic nerve agent appeared as C01-A040 (**12**) and the next A-234 (**3**): 0.68 and 0.71 mg/kg bw. Values between 1 and 1.55 mg/kg bw were achieved with compounds C01-A043 (**15**) and A-230 (**1**). Novichoks (**5, 11, 14** and **16**) pose a danger about 4–7 times lower than the structure mentioned above (**15**); they reach the following values: 7.35, 5.55, 4.38 and 5.04 mg/kg bw. Similar values were estimated for compounds (**13** and **17**); 15.35 and 15.49 mg/kg bw. ‘Iranian’ Novichok (**6**) and the compounds (**7**–**9**) showed values from 68.99 to 174.61 mg/kg, classifying them as one of the least toxic nerve agents studied. Ellison postulated the structure C01-A038 (**10**) at the LD_50_ value of 338.57 mg/kg bw, the highest result, and thus, the weakest of the Novichoks investigated.

Because the Consensus and FDA models showed numerous inaccuracies in the LD_50_ values for the Novichoks tested, we decided to additionally use the QSAR Toolbox to estimate the acute toxicity parameter. The results obtained by the subsequent software made it possible to verify the reliability of the calculations obtained from the Consensus and FDA models implemented in TEST by comparing the data. Acute toxicity values were estimated using the QSAR Toolbox for most compounds correlated with the Consensus method of the TEST software. Only two Novichoks (**2** and **6**) were more consistent with the FDA method. However, in the case of compounds (**10**–**11**) where the values for both TEST methods are comparable, a greater degree of correlation cannot be unequivocally determined. The assessed value for structure (**12**) in the QSAR Toolbox varies between the Consensus and FDA methods. A common feature of all estimations is the highest value achieved by Novichok C01-A038 (**10**). By analogy to the Consensus method, the first five compounds with the lowest LD_50_ values and thus posing the most significant threat are, respectively: A-232 (**2**), A-234 (**3**), A-230 (**1**), C01-A043 (**15**) and C01-A040 (**12**).

## Discussion

Based on sources from the available literature, the acute toxicity of Novichoks was perceived to be several times higher than that of conventional NAs (Ellison 2007; Mirzayanov 2008). Novichok A-230 was claimed to be 5–8 times more toxic than compound VX (a relative comparison of the LD_50_ values under the same conditions). Additionally, A-232 was said to be 10 times as harmful as Soman. Nerve agents A-242 and A-262, toxic derivatives of A-230 and A-232, were classified as ultra-highly toxic despite not specifying any value. However, these primary sources lack information on the acute toxicity of A-234 (Mirzayanov 2008). According to the data published in the seminar paper by Karev, the above reports on Novichok toxicological data were not valid (Karev 2009). In the case of A-232, it showed a value one order of magnitude lower than VX and A-234 two times lower than VX. The acute toxicity of the A series nerve agents, lower than conventional NAs, was somewhat confirmed by other estimated data (Franca et al. 2019). The LD_50_ value for Novichok A-230 was lower by an order of magnitude, while the compounds A-232 and A-234 were approximately three times less toxic than VX. It is worth mentioning that the similarity in toxicity between A-232 and A-234 was assumed here based on structural similarity. The results for these two Novichoks were highly different compared to the data published by Karev (2009).

Carlsen (2019) also attempted to verify these data for Novichoks (*n* = 6), using quantitative structure–activity relationship (QSAR) models for estimation and calculated the median lethal dose for oral administration rats that were converted to the human dose. Carlsen presented utterly different data on the relative toxicity of A-series nerve agents, contrary to Mirzayanov's claims. The estimated LD_50_ value for compound VX was used to indicate the reliability of the data. In the works of Karev (2009) and Franca et al. (2019), it reached the value of 10 mg/person (70 kg). Converted to mg/kg, this value is 0.14 mg/kg, which is very consistent with the calculated value of LD_50_ for humans: 0.1 mg/kg (Carlsen 2019). Estimated data proved that Novichoks (A-230, A-232, A-234, A-242, and A-262) are 5–75 times less dangerous than VX, and in the case of Iranian 'Novichok', almost 1000 times less toxic. While the values for A-232 and A-234 are similar to the data obtained by Franca et al. (2019), the median lethal dose for A-230 is very different. Assuming that a "standard" person weighs 70 kg, the LD_50_ values for Novichoks were recalculated based on the sources of the above literature and summarised in Table 4.

**Table 4:** Available literature data about the toxicity of Novichoks; based on (Karev 2009; Gupta 2015; Nepovimova and Kuca 2018; Franca et al. 2019; Carlsen 2019)

Compound	Human LD_50_ (mg/kg bw)		
	(Karev 2009; Nepovimova and Kuca 2018)	(Gupta 2015; Franca et al. 2019)	(Carlsen 2019)
VX	0,143	0.086–0.143	0.10
A-230	n/a	0.011–0.029	1.55
A-232	0.014	0.5	0.57
A-234	0.071	0.5	0.71
A-242	n/a	n/a	0.49
A-262	n/a	n/a	7.35
Iranian	n/a	n/a	96.16

*n/a*  not applicable

Using the analogous FDA method included in the TEST programme (ver. 4.2.1), we obtained precisely the same results for Novichoks as in the work of Carlsen (2019). Furthermore, to estimate the LD_50_ parameter, we also used the Consensus method included in TEST (ver. 5.1.2) and the second software, QSAR Toolbox. As the results between the Consensus and FDA methods differ, the question is which is more reliable? The first is supported by the fact that it applies all QSAR methods included in the TEST to assess toxicity and is additionally recommended by the US EPA (2022). Furthermore, the consensus method was reported to be the most reliable estimate provided by the TEST software (Melnikov et al. 2016). On the other hand, the FDA method is backed by the generation of new models based on the closest analogues of the test chemical. The latest TEST software version (ver. 5.1.2) does not include the implemented FDA method. Furthermore, the LD_50_ values obtained using the QSAR Toolbox overwhelmingly correlate with the consensus method; only two compounds from the A series nerve agents were the values more comparable to the FDA method. Taking into account the information above, we tend to evaluate the results obtained using the consensus method, primarily supported by verification using the QSAR Toolbox, as more trustworthy. The LD_50_ values for Novichoks estimated in our work using TEST (Consensus method) and QSAR Toolbox mainly differs from the data discussed earlier and are included in Table 4. The only compounds whose LD_50_ values were similar are A-230, A-232, and 'Iranian' Novichok. According to our estimates, the most dangerous Novichok was A-232, in contrast to the values calculated by Carlsen (2019), where the nerve agent A-242 would be the most toxic, and Franca et al. (2019) suggested A-230 as the most toxic Novichok. Unfortunately, sources from the literature only provide LD_50_ for 2–6 organophosphorus compounds from the Novichok group. Therefore, our work is unique because it includes up to 17 such A-series nerve agents.

Attention should also be paid to extrapolating doses between species, converting the rat to a human oral median lethal dose. The allometric scaling between species for dose conversion from animal to human studies is one of the most controversial areas of pharmacology and toxicology. The allometric approach considers differences in body surface area related to animal weight while extrapolating doses between species (Nair and Jacob 2016). This article's conversion to human toxicity followed the guidelines for animal-human dose conversion based on body surface area, i.e., rat doses were converted to human equivalent doses by dividing the rat dose by 6.2 (Nair and Jacob 2016; Carlsen 2019). Science changes in phases, experiencing anomalies that lead to a crisis and revolution, resulting in a new, immature scientific paradigm that, over time, becomes the new normal (Hartung 2021). Toxicology has encountered a series of such anomalies that have led to a crisis. One of them is the generally accepted guide for dose conversions between species, which is not necessarily the right one. As evidenced by the various studies, for example, many inflammatory mediators assume very different roles in different species; for example, TLR4 signaling differs in humans and mice (Schmidt et al. 2010). The above studies prove that animals are not particularly good predictors of humans in areas where we have comparative data across species. In toxicodynamics, a well-known example is that humans are 1000 times more responsive to inhibition of Na/K-ATPase by the cardiac glycoside ouabain than mice (Kent et al. 1987). Moreover, the difference in susceptibility to bacterial endotoxins can be up to a million times greater in range (Hasiwa et al. 2013). Thus, the above examples indicate that humans are not 70 kg mice in toxicology (Leist et al. 2008). A study based on a broad system approach confirmed the low predictability of animal responses to inflammation (Seok et al. 2013). The low-level predictability of animal studies in research areas, which allows direct comparison of data between species, raises serious doubts about the usefulness of animal data as crucial tools for predicting human safety. Perhaps this is the reason for the differences in prediction, or perhaps it is another proof of the validity of Hartung's concept? (Hartung 2009, 2021). Regardless, these studies were essential as an initial screening before undertaking acute toxicity studies on animals, concerning reactive substances such as Novichoks.

## Conclusions

Undoubtedly, Novichoks pose a grave threat to human security. We have had the opportunity to experience examples of their excessive toxicity three times, including in Salisbury, Amesbury (UK) and the case of Navalny's poisoning. Some light was shed on the acute toxicity of Novichoks by estimating the median lethal dose (LD_50_) of these hazardous nerve agents. The estimation has been made for organophosphorus compounds from the Novichok group using in silico tools: Toxicity Estimation Software Tool (TEST) and QSAR Toolbox. According to our evaluations, the deadliest Novichoks were compound A-232 (**2**), A-230 (1) and A-234 (**3**), whose LD_50_ values, when administered orally, did not exceed 0.65 mg/kg bw. On the other hand, the 'Iranian' Novichok (**6**) and C01-A038 (**10**) compounds, whose values exceeded 130 mg/kg bw, proved the least perilous. Unfortunately, despite the update of the CWC list, the exact structure of Novichoks is not known. It should be emphasised that the complete threat posed by Novichoks, in addition to the toxicity itself, also includes processes such as the vapour pressure, water solubility, skin permeability coefficients, the toxicokinetics, and the environmental fate of these compounds, which determine their durability in the external environment. Further in silico studies of different properties (chemical, physical, and toxicological) are required to deal with the inevitable utilisation of novel types of nerve agents in terrorist attacks. Our toxicology studies provide the first comprehensive insight into the acute toxicity of numerous Novichoks (*n* = 17). The TEST and QSAR Toolbox software can be successfully applied as tools to estimate the median lethal dose of organophosphorus compounds of the Novichok group preceding experimental laboratory tests.

## Data Availability

All data generated or analysed during this study are included in this published article.

## Abbreviations

ACh: Acetylcholine
AChE: Acetylcholinesterase
bw: Body weight
CWC: Chemical Weapons Convention
LD_50_: Median lethal dose
NA: Nerve agent
NATO: North Atlantic Treaty Organization
OP: Organophosphate
OPCW: Organization for the Prohibition of Chemical Weapons
QSAR: Quantitative structure–activity relationship
SMILES: Simplified Molecular Line Input System
TEST: Toxicity Estimation Software Tool
